# Modeling Bilingual Lexical Processing Through Code-Switching Speech: A Network Science Approach

**DOI:** 10.3389/fpsyg.2021.662409

**Published:** 2021-08-25

**Authors:** Qihui Xu, Magdalena Markowska, Martin Chodorow, Ping Li

**Affiliations:** ^1^Department of Psychology, Graduate Center, The City University of New York, New York, NY, United States; ^2^Department of Linguistics, Institute for Advanced Computational Science, Stony Brook University, Stony Brook, NY, United States; ^3^Department of Psychology, Hunter College, The City University of New York, New York, NY, United States; ^4^Department of Chinese and Bilingual Studies, Faculty of Humanities, The Hong Kong Polytechnic University, Hong Kong, China

**Keywords:** code-switching speech, bilingual lexicon, network science, community detection, clustering coefficient, computational linguistics

## Abstract

The study of code-switching (CS) speech has produced a wealth of knowledge in the understanding of bilingual language processing and representation. Here, we approach this issue by using a novel network science approach to map bilingual spontaneous CS speech. In Study 1, we constructed semantic networks on CS speech corpora and conducted community detections to depict the semantic organizations of the bilingual lexicon. The results suggest that the semantic organizations of the two lexicons in CS speech are largely distinct, with a small portion of overlap such that the semantic network community dominated by each language still contains words from the other language. In Study 2, we explored the effect of clustering coefficients on language choice during CS speech, by comparing clustering coefficients of words that were code-switched with their translation equivalents (TEs) in the other language. The results indicate that words where the language is switched have lower clustering coefficients than their TEs in the other language. Taken together, we show that network science is a valuable tool for understanding the overall map of bilingual lexicons as well as the detailed interconnections and organizations between the two languages.

## Introduction

Bilinguals frequently alternate between two languages in their daily life, a phenomenon often referred to as code-switching (CS). In conversations between interlocutors of similar bilingual backgrounds, CS speech can be widely observed within a single discourse or even the same sentence (Poplack, 1980). A growing number of studies have found unique processes involved in free and voluntary language switching of words in contrast to involuntary switching under cued instructions in experiments (Gollan and Ferreira, 2009; Gollan et al., 2014; Gross and Kaushanskaya, 2015; Kleinman and Gollan, 2016; Blanco-Elorrieta and Pylkkänen, 2017; de Bruin et al., 2018). Unlike involuntary CS, voluntary CS does not necessarily incur switching cost (Li, 1996; Grosjean, 1997; Blanco-Elorrieta and Pylkkänen, 2017), and it is affected by lexical accessibility (Gollan and Ferreira, 2009; Gollan et al., 2014; Gross and Kaushanskaya, 2015; Kleinman and Gollan, 2016; de Bruin et al., 2018). These studies together underscore the importance of understanding CS speech as well as the implications of CS speech for bilingual language representations.

The present study asks (a) how CS processes reflect lexical representations of different languages and (b) what potential factors affect bilinguals’ CS behaviors. Instead of treating words as independent of each other, as in most previous studies, we examine the research questions through a novel method drawn from network science analyses, specifically, by probing the mutual connections and interactions in the semantic structure of bilingual lexicons based on bilingual CS speech production.

### Language Representations Reflected in CS Speech

Understanding how bilinguals represent and organize lexicons in two languages has long been a fundamental area of research in bilingualism. The Competition Model, an emergentist theory of language processing and acquisition, proposes a competitive interplay between the two languages that allows bilinguals to organize multiple languages without massive interference (Bates and MacWhinney, 1982). Separate modular representations for different languages can be constructed or emerge out of the processes of lexical or grammatical competition (Hernandez et al., 2005; Hernandez, 2013), as bilinguals use language-specific cues and within-language resonance during language usage.

Traditionally, studies have examined the emergentist theory of language processing by cued experimental tasks that involve explicit interventions (e.g., Costa and Santesteban, 2004; Rubin and Meiran, 2005). When bilinguals are only allowed to switch between languages following an experimenter-supplied cue, they usually experience a cognitive cost during switching and therefore take longer to complete the experimental task. The widely observed switching cost reveals important language representation mechanisms, such as a co-activation of words in different languages and a language control system that monitors which language to produce (Dijkstra and Van Heuven, 2002), which are consistent with the emergentist view that separate lexical modules arise from the competitive interplay between languages (Hernandez et al., 2005; Hernandez, 2013).

Nevertheless, some studies have challenged the switching cost phenomenon by showing that voluntary CS speech, in which bilinguals are free to choose either language to produce, can be cost free (Li, 1996; Gollan et al., 2014; Gross and Kaushanskaya, 2015; Kleinman and Gollan, 2016; Blanco-Elorrieta and Pylkkänen, 2017; de Bruin et al., 2018). Blanco-Elorrieta and Pylkkänen (2017) found that voluntary CS speech in spontaneous conversations did not engage the pre-frontal cortex, a brain region known for language control (Crinion et al., 2006), or induce any behavioral costs. Kleinman and Gollan (2016) specifically characterized the benefit of voluntary switching on production. While involuntary switching elicited a switch cost compared to staying in one language, participants showed enhanced performance in picture naming when being allowed to freely switch between languages. As growing evidence suggests that voluntary CS might involve different cognitive processes from CS traditionally studied under involuntary conditions, it is important to revisit the language representation mechanisms through voluntary CS in spontaneous conversations.

Apart from behavioral studies with CS tasks, neurolinguistic research has provided a different perspective of the emergentist theory of bilingual language representation. A large body of neuroimaging studies has shown that different languages are organized in a shared brain system (e.g., Klein et al., 1995; Chee et al., 1999; Perani and Abutalebi, 2005). However, there has also been research showing distinct brain activities associated with different languages (Dehaene, 1999; Li, 2009; Xu et al., 2017). Xu et al. (2017) observed different activity patterns in the brain when Mandarin-English bilinguals were processing different languages. They further argued that different languages might engage interleaved, but functionally independent, neural populations, although those populations might be located in the same cortical areas. The mixed findings (e.g., Chee et al., 1999; Xu et al., 2017) raise concern of a lack of fine-grained information from studies relying on neuroimaging data.

While it is important to seek a clear picture of bilingual language representation, the heterogeneity and diversity of bilingualism is an important factor to be considered. Bilinguals live in different environments and can vary along many dimensions, such as the age of acquisition, language proficiency, and the relative distance between L1 and L2, to name a few. The age of acquisition and language proficiency have long been identified as key factors that give rise to neural and cognitive variations in bilinguals (Hernandez, 2013; Li, 2013a), but the relative distance between the two languages that bilinguals speak has received less attention until only recently (Li, 2013b; Abutalebi et al., 2015; Kim et al., 2016; Ramanujan, 2019a,b). Several studies have reported different brain structures between bilinguals speaking two linguistically closer languages and those who speak two linguistically distant languages (Abutalebi et al., 2015; Kim et al., 2016; Ramanujan, 2019a). Ramanujan (2019b) found that Dutch-English, two linguistically closer languages require greater cognitive control than Cantonese-English, whose linguistic attributes have greater disparities. The relative distance between languages was found to play a critical role in bilingual language representation in these recent studies.

Taken together, the existing literature is mixed with regard to the nature of bilingual lexical representation. A study of spontaneous bilingual CS speech could shed new light on this topic. If bilinguals can frequently and automatically retrieve words from two languages, does that still support the emergent lexical modules between languages as previously suggested (e.g., Hernandez et al., 2005; Hernandez, 2013)? If bilingual language processing can be shaped by the relative distance between the two languages, will the lexicons differ among different bilingual groups? The current study is aimed at examining these questions.

### Code-Switching and Lexical Accessibility

Assuming that the emergentist view of modular representations of languages is correct, another question that arises is why bilinguals can often switch back and forth between the two language modules with no apparent cognitive cost. Research has consistently shown that the accessibility of words may account for language choice during spontaneous CS speech (Gollan and Ferreira, 2009; Gollan et al., 2014; Gross and Kaushanskaya, 2015; Kleinman and Gollan, 2016; de Bruin et al., 2018). Bilinguals choose to switch languages when the word in the other language is more accessible than the equivalent word in the current language.

Researchers have used a variety of methods to measure lexical accessibility, including presenting words with different frequencies in the two respective languages (Gollan and Ferreira, 2009; Gross and Kaushanskaya, 2015) or with different levels of subjective familiarity (Gollan et al., 2014), and measuring reaction times in picture naming tasks (de Bruin et al., 2018). Among these accessibility measurements, word frequency has often been examined in bilingual CS studies (Gollan and Ferreira, 2009; Gross and Kaushanskaya, 2015). Gollan and Ferreira (2009) found that English-dominant bilinguals tended to choose the non-dominant language when pictures had high-frequency names in both languages, whereas pictures with low-frequency names were more likely to be named in the dominant language. Gross and Kaushanskaya (2015) observed similar patterns in bilingual children who were more likely to name pictures in their non-dominant languages if the picture names were highly frequent and early acquired words in both languages. However, the link between frequency and language choice in CS speech is more nuanced. Some studies noted that the items named in the non-dominant language did not necessarily have lower frequency in the dominant language; rather, they were highly frequent in both languages in general (Gollan and Ferreira, 2009; Gross and Kaushanskaya, 2015). de Bruin et al. (2018) did not observe any association between frequency and language choice.

Despite the significance of lexical accessibility for language switching, some important aspects have been overlooked by the previous studies. First, the evidence mainly comes from picture naming studies of word-by-word switching, which might differ from CS speech in natural conversations. Unlike producing a set of unrelated words one at a time, words produced by bilinguals in natural settings are connected within the context of the sentence or discourse. Second, almost all previous studies of lexical accessibility focused on a local rather than a global context. A local context treats words as being independent of each other, whereas a global context considers the interconnections between words in a dynamic way (Karuza et al., 2016). Evidence has emerged that the architecture of the word’s global system (i.e., overall lexical-semantic organization) can also affect the retrieval of the word during speech recognition and production (Chan and Vitevitch, 2009, 2010). However, in the domain of bilingual CS speech, little has been done to understand how the global structure of an interconnected lexical system can affect lexical retrieval.

### Network Science Approach to Language Processing

Recently, network science has become an important domain of study across interdisciplinary research in psychology, linguistics, and neuroscience (e.g., Chan and Vitevitch, 2010; Bassett and Sporns, 2017; Karuza et al., 2017, 2019; Sizemore et al., 2018; Tiv et al., 2020) and has also been increasingly applied to understanding language representation and processing (Steyvers and Tenenbaum, 2005; Chan and Vitevitch, 2009, 2010; Hills et al., 2009; Sizemore et al., 2018). The methodology is powerful in capturing not only the global architecture of a complex system as a whole, but also the detailed interaction patterns between different pieces of information. A substantial number of studies have suggested the influence of network structure on many aspects of language processing (for a review, see Karuza et al., 2016).

**Figure 1:**
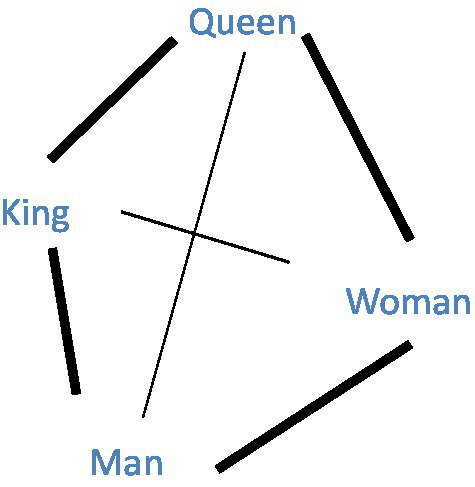
An illustration of a weighted semantic network with four words. Each node represents a word. Each edge is the link between two words that represents the semantic association between those words. The edge weight denotes the semantic similarity between the two linked nodes, which is represented by the thickness of the line in the figure. For example, the similarity between “Queen” and “Woman” is greater than the similarity between “Queen” and “Man.”

The present work uses semantic networks (Steyvers and Tenenbaum, 2005), one important type of network wherein words are organized based on their semantic meanings. In a weighted semantic network, unique word types are represented as *nodes*. Semantic associations between two words are represented as weighted *edges*, reflecting the strength of the semantic association between the two words. Figure 1 illustrates a weighted semantic network. With nodes in various connection patterns, the topological structure of a semantic network can indicate unique properties of lexical representations. Given our research aims to investigate semantic organization of words in two languages and the factors affecting bilingual CS, we focused on two measurements in network science, i.e., community and clustering coefficients.

#### Community

Community refers to a group of nodes that are more densely connected to each other than with the rest of the nodes of the network. Multiple algorithms have been proposed for detecting communities in topological networks. Among them, the Louvain algorithm (Blondel et al., 2008) has been shown to outperform other algorithms in the previous research (Lancichinetti and Fortunato, 2009; Yang et al., 2016). The Louvain algorithm (Blondel et al., 2008) detects communities by optimizing modularity value (Q), a value defined as the relative density of edges inside communities compared to edges outside communities (Newman and Girvan, 2004). The Louvain algorithm repeatedly includes nodes in the community that yield the largest increase in modularity, until the modularity value no longer increases. For more information about the Louvain algorithm, see Blondel et al. (2008).

Community is considered an important structural property in network science as it helps discover the internal relationships between nodes at a global level (Yang et al., 2016; Tiv et al., 2020). Studies have shown that participants are sensitive to community structures (Karuza et al., 2017, 2019). For example, Karuza et al. (2017) asked participants to process a sequence of images generated based on a modular network with three communities. They found that participants’ processing time sharply increased when the stimulus was shifted from images in one community to images in a different community. The observed pattern was further replicated in Karuza et al. (2019) by showing that the processing cost caused by between-community shift was robust even when the topological structure of the network, such as network size and number of communities, was varying. The findings together signify the association between community structures and human information processing.

#### Clustering Coefficients

**Figure 2:**
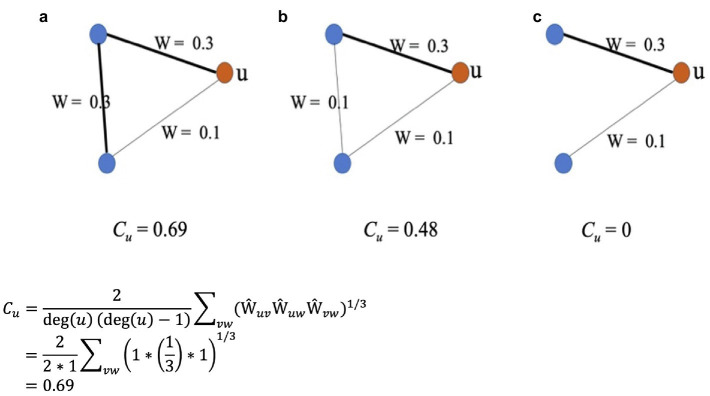
An illustration of the clustering coefficient, *C_u_*, of a node *u* in three different weighted networks: a, b, and c. The three networks have an equal number of nodes but differ in edge weights. Also shown is the detailed calculation of *C_u_* for network a.

The clustering coefficient measures the probability that neighbors of a node are themselves neighbors (Watts and Strogatz, 1998), much as in social networks, where close friends are often friends with similar groups/clusters of people. In the case of a semantic network, the clustering coefficient of a word represents the extent to which a word’s semantically similar words are also similar to each other. It reflects how clustered (i.e., grouped together) the semantic representations are for the word and its semantically similar words. As shown in Equation (1), the clustering coefficient of a node in a weighted network is calculated by taking the sum of the geometric average of the edge weights of that node (Onnela et al., 2005). In Equation (1), *deg(u)* denotes the node’s degree, which represents the number of edges a given node is connected to. *Ŵ_uv_*, *Ŵ_uw_*, and *Ŵ_vw_* are the weights of the three edges between the node and its two neighbors, which are normalized by the maximum weight in the network as shown in Equation (2). For an illustration of the calculation, see Figure 2.


(1) $$C_{u}=\frac{2}{\deg\left(u\right)\left(\deg\left(u\right)-1\right)}\sum_{vw}{\left({\hat{W}}_{uv}{\hat{W}}_{uw}{\hat{W}}_{vw}\right)}^{1/3}$$

(2) $$\hat{W}=\frac{W}{\max\left(w\right)}$$

Previous research in phonological networks has shown the influence of clustering coefficients on spoken word recognition and production (Chan and Vitevitch, 2009, 2010). Chan and Vitevitch (2010) examined this influence through both corpus analysis and experimental tasks. The corpus analysis suggested that speech errors were more likely to occur in words with higher clustering coefficients. The experiment’s results replicated that pattern in that participants spent a longer time on naming pictures representing words with higher clustering coefficients (e.g., “bash,” “bag,” and “bad”) than words with lower clustering coefficients (e.g., “log,” “league,” and “leg”). Chan and Vitevitch (2010) explained the observed pattern by proposing that a word with fewer interconnected neighbors (i.e., lower clustering coefficient) can more easily “stand out” among other similar words. In contrast, a word with densely interconnected neighbors (i.e., higher clustering coefficient) is less distinctive and hence is more difficult to retrieve from many other words with similar sounds. However, it remains unclear whether the clustering coefficient can capture properties of semantic networks as it does in phonological networks, and whether lower clustering coefficients within semantic networks could also similarly facilitate lexical accessibility in speech production.

### The Present Study

The present research investigates CS processes from the perspectives of representation and accessibility. To gain a deeper understanding of bilingual language representations underlying CS, we constructed semantic networks on CS speech corpora and conducted community detections to depict the semantic organizations of the bilingual lexicon. If spontaneous CS speech reflects separate lexical modules between languages, we would expect that words from different languages would largely reside in different communities within the semantic network, at least for proficient adult bilingual speakers. To examine whether the spontaneous CS behaviors are affected by lexical properties in an interconnected semantic system, we compared clustering coefficients of words that were code-switched with their translation equivalents in the other language. We predict that the clustering coefficient property in the semantic domain plays a role similar to the one it plays in the phonological domain (Chan and Vitevitch, 2010). That is, the clustering coefficient of a word is related to the likelihood of its being code-switched. Specifically, we hypothesize that a word that is code-switched, the word produced in a language different from the preceding word, will have a lower clustering coefficient than its translation equivalent in the counterpart language that is replaced. To explore potential impacts of cross-language differences, we examined two different groups of balanced bilinguals, Mandarin-English bilinguals and Spanish-English bilinguals.

## Study 1

Study 1 established the semantic organizations of the bilingual lexicon by building semantic networks on CS speech. We first trained word embedding models (Mikolov et al., 2013; Bojanowski et al., 2017) to obtain semantic associations between words. A weighted semantic network was then constructed for each bilingual group, with words being nodes and semantic similarities obtained from the embedding models being edge weights. Community detections (Blondel et al., 2008) were conducted to detect existing groupings. Finally, we depicted the semantic organizations of bilingual lexicons by analyzing the proportions of words from two languages within each community.

### Methods

#### Materials

In selecting bilingual CS speech corpora to analyze, we focused on the language pairs of Mandarin-English and Spanish-English. The two language pairs have been commonly studied in CS research, with sizable and publicly accessible data (see Sitaram et al., 2019 for a review of all the available CS speech data). Using English as the common language and Mandarin vs. Spanish as the other language in the pair, we have the advantage of examining both similarities and differences across bilingual populations. We used the corpus of Mandarin-English CS in southeast Asia (SEAME; Lee et al., 2017) for the Mandarin-English data and the Bangor-Miami corpus (Deuchar et al., 2014) for the Spanish-English data.

##### Mandarin-English CS in Southeast Asia

Mandarin-English CS in southeast Asia (Lee et al., 2017) includes free conversations and interviews with 99 subjects from Singapore and 58 subjects from Malaysia. To have some consistency in the speech content and the subjects’ demographics, we focused on free conversations of Singaporean participants. Singapore has a bilingual language policy where English is the official working language used at school and in the communities where they live, and Mandarin is the official mother tongue for the Chinese population (accounting for 74.3% of the Singaporean population, according to the Singapore Department of Statistics, 2014). Therefore, all the Mandarin-English bilingual subjects are expected to be proficient in both languages. On the other hand, English has been viewed as the more dominant and widely used language according to many studies (Zhao et al., 2007; Singapore Department of Statistics, 2014; Curdt-Christiansen and Sun, 2016; Sun et al., 2018).

Although the corpus did not provide language identities for the words, we could easily detect the language of the words in the Mandarin-English corpus based on their encodings, as Mandarin words and English words were encoded by different sets of unicode characters (Aliprand, 2011). Data were preprocessed by excluding non-word markers (e.g., “<unk>”) and words that are communicators (e.g., “eh” and “orh”). As there is no natural word boundary in Mandarin (e.g., spacing as in English), accurate word segmentation is necessary in dividing text into words. Although the original data had relied on automatic word segmentation and manual checking, we noticed that many segments still contained more than one word. For example, the segment “我不知道” (i.e., “I do not know”) should have been further segmented into words “我” (“I”), “不” (“not”), and “知道” (“know”). Therefore, we re-applied word segmentation with PKUSEG (Luo et al., 2019), a state-of-the-art segmenter with F-score as high as 96.88, indicating a high degree of accuracy and recall for word segmentation. After preprocessing, there were 58,534 sentences in the analysis, with 6,986 unique words in Mandarin and 6,734 unique words in English.

##### Bangor Miami

This corpus contains bilingual speech from Spanish-English speakers living in Miami, the United States (Deuchar et al., 2014). Most bilinguals in Miami use Spanish at home but learn and use English in school and their community. Most of the subjects in the corpus (Deuchar et al., 2014) reported high proficiency and equally frequent use of the two languages, with English being more dominant according to the language background information of the subjects (Deuchar et al., 2014).

The corpus has manually annotated language identity for each word. Data were preprocessed: Punctuations were removed. Words that are neither English nor Spanish words were removed, which accounted for less than 1% of all words in the corpus. After preprocessing, there were 45,610 sentences in the analysis, with 6,308 unique words in Spanish, 6,939 unique words in English, and 1,727 unique words labeled as “English or Spanish” – words with mixed morphemes from the two languages, words that are proper nouns (e.g., “Popeye,” a cartoon character), or words whose pronunciations are identical between the two languages (e.g., “no”).

##### Code-Switching Types

There are three major types of sentences in bilingual CS speech: sentences that do not involve any switches (non-CS sentences), sentences that have words from different languages (intra-sentential CS sentences), and sentences that do not involve intra-sentential switches but are in a language that is different from that of the immediately preceding sentence (inter-sentential CS sentences). Given that some words in the Spanish-English corpus have ambiguous language identity, we adopted a conservative rule such that only words with clear language identity (unambiguous words) would be used for classification. Namely, a sentence needs to contain unambiguous words in both English and Spanish in order to be counted as an *intra-sentential* CS sentence. Similarly, an *inter-sentential* sentence must contain unambiguous words in a language different from that of its immediately preceding sentence. Table 1 presents examples of the three types of sentences.

**Table 1:** Examples of the three CS types in bilingual speech.

CS type	Mandarin-English	Spanish-English
Non-CS	-Are you sure?	-Fine you do not want me to do
	-**Actually I do not think that’s necessary.**	eng eng eng eng eng eng eng
		yours ok.
		eng eng&spa
		-**Because I need to submit it online by Monday.**
		eng eng eng eng eng eng eng
		eng eng
Intra-sentential CS	- 刚才 我 不是 跟 你 讲 我 apply 那个 job?	**-Ok un beso a ella también bye bye.**
	(Haven’t I told you just now that I applied for that job?)	eng&spa spa spa spa spa spa
		eng eng
		(Ok a kiss to her too bye bye.)
Inter-sentential CS	-我 要 回家 看。	-a ver.
	-**It was exciting once.**	spa spa
		-**She likes Pam too**.
		eng eng eng&spa eng

*Highlighted sentences are examples of non-CS sentences, intra-sentential CS sentences, and inter-sentential CS sentences. For the non-CS sentences and the inter-sentential CS sentences, their immediately preceding sentences are also presented. Translations in English are provided for the intra-sentential CS sentences. In the Spanish-English examples, “spa” represents Spanish, “eng” represents English, and “spa&eng” represents ambiguous words that can be in either Spanish or English*.

#### Semantic Networks and Word Embedding Models

To obtain an overall representation of bilingual semantic lexicons, we built a weighted semantic network on the whole corpus for each bilingual group. As the current research primarily focuses on word-level CS, and the intra-sentential CS sentences are where the word-level language mixing occurs, for each bilingual group we established additional semantic networks for the intra-sentential CS sentences only. For each network, the nodes were from all unique words of the sentences being included. The edge weight between each pair of nodes was determined by the semantic association between those two nodes, which was obtained from semantic vectors as follows.

##### Semantic Vectors

Word embedding, a technique for capturing the distributional properties of words embedded in large stretches of sentences and discourses, was used to train the semantic vector of each individual word. There are many different word embedding models that use large-scale distributions of text or discourse. Among them, word2vec (Mikolov et al., 2013) and fastText (Bojanowski et al., 2017) are two well-accepted models with similar algorithms but different feature representations. Word2vec is an artificial neural network model widely used in corpus linguistics and natural language processing that learns vector representations of words from text. Words that appear in similar contexts are closer in vector space. Because semantically related words tend to exist in similar contexts (e.g., “king” and “queen”), word2vec can well capture semantic associations between words (Mikolov et al., 2013). For example, it can derive word vectors that display semantic similarities between word pairs, such that the semantic association between “queen” and “woman” is analogous to the association between “king” and “man.”

The implementational algorithm of fastText (Bojanowski et al., 2017) is similar to word2vec, except that fastText learns vector representations of character n-grams rather than words; vectors of words are the sum of the n-grams they are made of. Therefore, fastText can represent the semantic meanings of words with fine-grained sublexical information, such as morphemes in English and radicals in Mandarin characters.

Studies have shown that fastText outperforms word2vec on representing semantic meanings of words perhaps because of the incorporation of sublexical information (Bojanowski et al., 2017; Grave et al., 2018). Given that, Study 1 primarily used the fastText model to obtain semantic vectors for words. However, one potential risk of using fastText is that the sublexical features might overestimate connections of words within the same language. For example, the vectors of English words are all made from English morphemes, whereas the vectors of Mandarin words are all made from Mandarin characters, which enlarges similarities of words within the same language. To rule out the possibility that the detected language separation, if there is any, is solely due to the cross-linguistic difference in sublexical features, we also constructed semantic vectors based on the word2vec model.

During training, the hyperparameters for deriving word semantic vectors were chosen based on the literature (Mikolov et al., 2013; Bojanowski et al., 2017) and were identical across the models of different corpora. For example, the dimensionality was 300, and the window size was five.

##### Edge Weights

The edge weight between two given words was obtained from the cosine similarity between those two words’ vectors. Cosine similarity is a measurement of the cosine of the angle between two vectors, which is widely used in representing the semantic similarity between any two words given the words’ embedding vectors (Mikolov et al., 2013; Bojanowski et al., 2017). To make our models computationally tractable and efficient, words with negative cosines, which represent high dissimilarity between the two words, were not connected.

#### Analysis

We first analyzed the frequencies and the proportions of non-CS, intra-sentential CS, and inter-sentential CS sentences out of all sentences in the bilingual speech. Next, for each bilingual group, we constructed semantic networks with variations in sentence types (i.e., all sentences or only the intra-sentential CS sentences) and embedding models (i.e., fastText or word2vec). Community detections (Blondel et al., 2008) were then run on each semantic network. Finally, with communities detected, we analyzed the proportions of words in each language within each community to test whether the two languages reside in different communities. As previously discussed (Community), nodes that reside in the same community are more densely connected with one another (i.e., forming stronger modularity) than nodes outside the community, and therefore, community is an important network metric for us to determine the global structure of lexical items.

### Results

In Mandarin-English bilingual speech, the proportions of non-CS sentences, intra-sentential CS sentences, and inter-sentential CS sentences were 41.2, 54.1, and 4.7%, respectively. In Spanish-English bilingual speech, however, the non-CS sentences accounted for 83.3% of all sentences, whereas the proportions of intra-sentential and inter-sentential sentences were 6.2 and 10.5%. For detailed statistics on sentences and word tokens of each CS type, see Table 2.

**Table 2:** Frequencies and percentages of sentences and word tokens of the three CS types in bilingual speech.

CS type	Mandarin-English	Spanish-English
Non-CS		
Sentences	24,119 (41.2%)	37,980 (83.3%)
*L1 (%)*	64.1	66.9
*L2 (%)*	35.9	30.3
Intra-sentential CS		
Sentences	31,676 (54.1%)	2,824 (6.2%)
*L1 (%)*	40.9	43.7
*L2 (%)*	59.1	54.0
Inter-sentential CS		
Sentences	2,739 (4.7%)	4,806 (10.5%)
*L1 (%)*	50.3	46.3
*L2 (%)*	49.7	51.0

*Sentences: the frequency and the percentage of sentences by CS type. L1(%): the percentage of word tokens in English; L2(%): the percentage of word tokens in Mandarin or Spanish out of all word tokens in sentences of the CS type. In the Spanish-English bilingual group, the proportions of word tokens with ambiguous language identities are not presented in the table; that is also why L1(%) and L2(%) do not add up to 100*.

**Figure 3:**
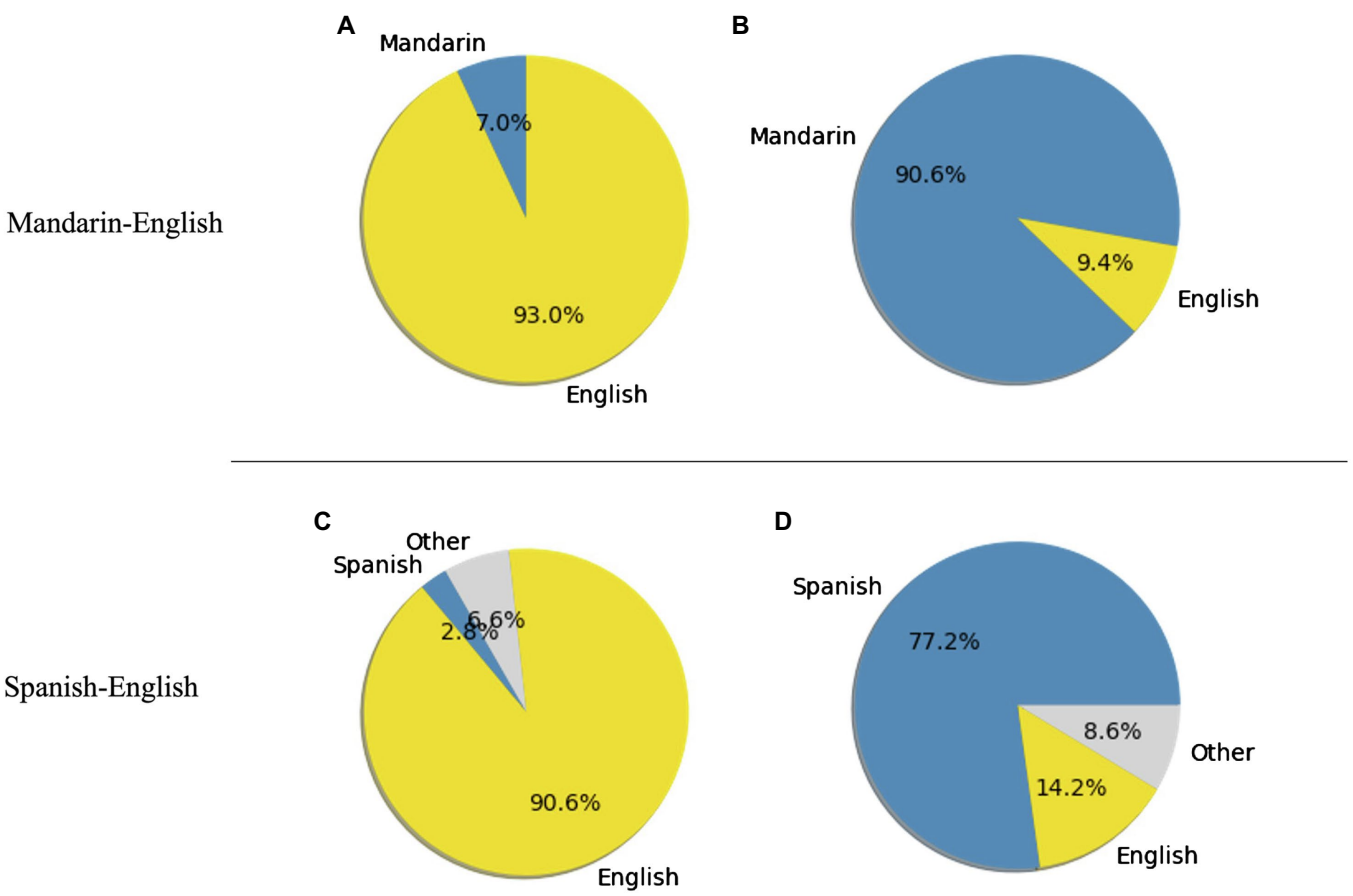
Proportions of words within each community in Mandarin-English (A,B), and Spanish-English (C,D). Networks are based on all sentences and word2vec embeddings. For the Spanish-English plots, “Other” represents words with ambiguous language identities.

Table 3 presents detailed community detection output. For the networks based on all sentences, the Louvain algorithm consistently detected two communities for each language pair. For each network, an analysis of the words’ language identity reveals that each community was dominated by one particular language. The dominance was greater for the community where English was the major language; that is, the proportion of words in English out of all words in the English-dominant community was greater than the proportion of words in Spanish or Mandarin in the other community (see Figure 3 for an illustration of the word2vec version of the network).

**Table 3:** Community detection output.

Sentence type	Bilingual group	Embedding	Community 1			Community 2			*Q*
			*N*	*L1(%)*	*L2(%)*	*N*	*L1(%)*	*L2(%)*	
All sentences	Mandarin-English	FastText	2846	99.4	0.6	2220	9.4	90.6	0.21
		Word2vec	2424	93.0	7.0	2642	29.6	70.4	0.06
	Spanish-English	FastText	1872	92.1	1.8	1708	11	79.7	0.17
		Word2vec	1838	90.6	2.8	1742	14.2	77.2	0.06
Intra-sentential CS only	Mandarin-English	FastText	2155	96.8	3.2	1907	19.8	80.2	0.09
		Word2vec	1810	87.1	12.9	2252	39.4	60.6	0.04
	Spanish-English	FastText	682	50.3	47.4	–	–	–	–
		Word2vec	682	50.3	47.4	–	–	–	–

*N: the number of words within the community; L1(%): the percentage of words in English; L2(%): the percentage of words in Mandarin or Spanish; and Q: the modularity value; see Section Community for the definition of modularity. In the Spanish-English bilingual group, the proportions of words with ambiguous language identities are not presented in the table; that is also why L1(%) and L2(%) do not add up to 100*.

**Figure 4:**
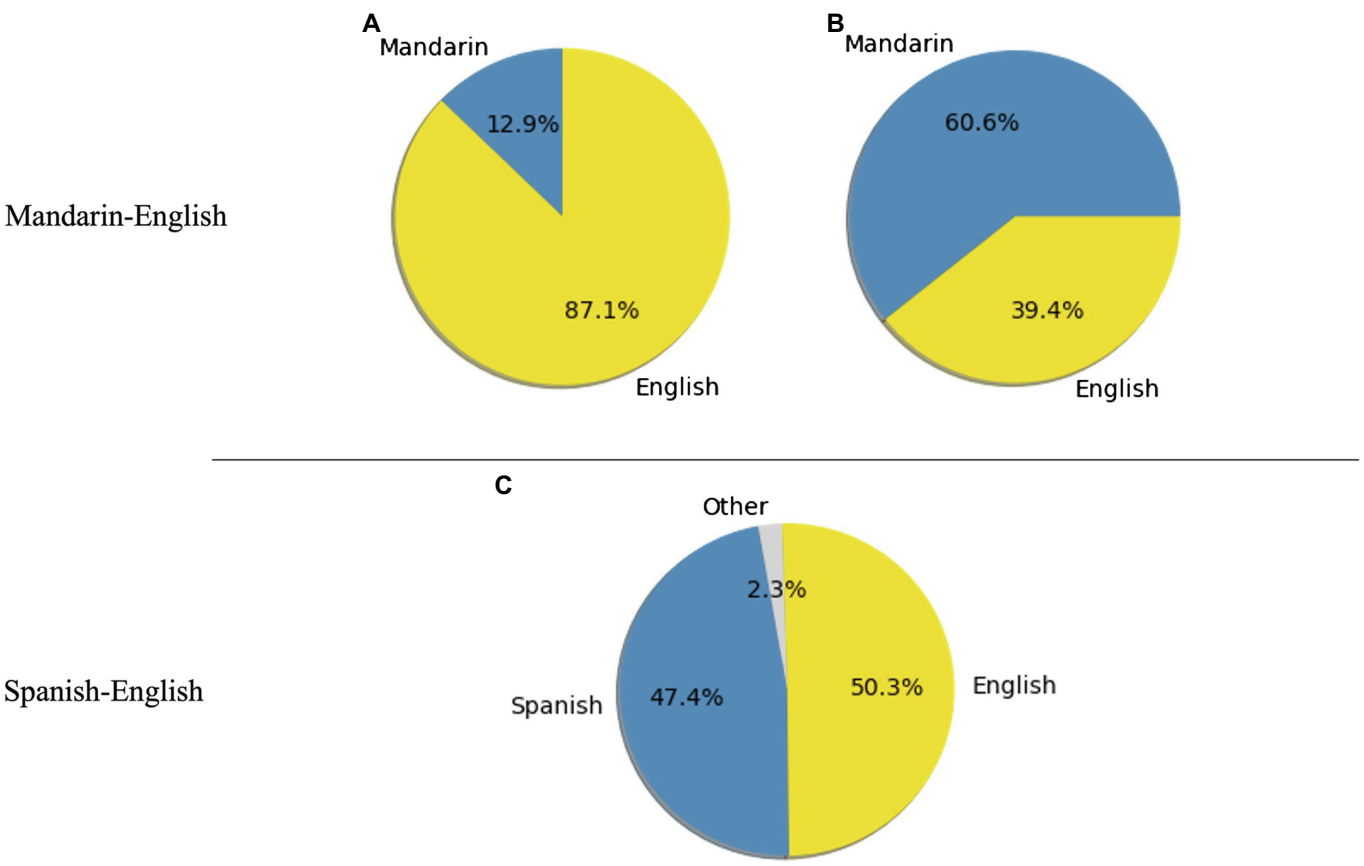
Proportions of words within each community in Mandarin-English, **(A)** and **(B)**, and Spanish-English, **(C)**. Networks are based on intra-sentential sentences and word2vec embeddings. For the Spanish-English plots, “Other” represents words with ambiguous language identities.

Focusing on the networks based on intra-sentential CS sentences only, the community detection output two communities for the Mandarin-English networks but only one community for the Spanish-English networks. For Mandarin-English networks, each of the communities was dominated by one particular language, and the dominance was again greater for the community where English was the major language (Figure 4).

### Discussion

We observed a consistent pattern between Mandarin-English and Spanish-English bilingual groups such that, based on the overall spontaneous CS speech, the two languages largely reside in separate communities. It suggests that bilinguals might have separate lexical modules in organizing semantic meanings of words. Such a pattern remains true for the Mandarin-English network, even when considering only the intra-sentential CS speech. Although there was just one detected community in the Spanish-English network based on intra-sentential CS sentences, the intra-sentential CS speech accounts for only a small portion of the total speech. Therefore, the overall findings are still in favor of two largely separate lexical modules in bilingual semantic representation. When separate communities were detected, the dominant language, English, showed greater dominance in the community compared to the non-dominant language in the other community. While the community with English as the major language leaves little space for words in the non-dominant language, the community with dominant Mandarin or Spanish is more open to English words.

Despite the overall pattern revealed by community analyses, there still exist different characteristics of CS speech between Mandarin-English and Spanish-English bilinguals. First, Spanish-English speakers code-switched less often than Mandarin-English speakers. Most of the sentences produced by Spanish-English speakers hardly involve any inter-sentential or intra-sentential CS. Second, unlike the Mandarin-English network, the Spanish-English network based on the intra-sentential CS speech showed only one integrated community.

Given that the observed semantic organizations of lexicons in the two languages are largely separate, it brings us to another important question: what drives bilinguals’ frequent switching back and forth between the two language modules in daily life? Is there any fundamental difference between the words being switched and the words being replaced? Study 2 addressed those questions with a focus on the interconnection and interaction between words.

## Study 2

This study examines whether the clustering coefficients of words capture important psycholinguistic properties that affect bilingual CS speech. Given the evidence that CS is related to lexical accessibility and that the clustering coefficient of a word reflects lexical accessibility in CS production (Chan and Vitevitch, 2009, 2010; Gollan and Ferreira, 2009; Gollan et al., 2014; Gross and Kaushanskaya, 2015; Kleinman and Gollan, 2016; de Bruin et al., 2018), we predicted a similar role of clustering coefficients on the choice of language during bilingual CS speech. By comparing clustering coefficients of words that were code-switched (CS words, i.e., words that were produced in a language different from the preceding word) with their TEs in the other language (TEs, i.e., words in the preceding language that were replaced), we hypothesized that the CS words have lower clustering coefficients than their TEs. In addition, word frequency, a traditional indicator of lexical accessibility, was considered and controlled for in evaluating the effect of clustering coefficients.

### Methods

#### Materials

##### Code-Switching Words and TEs

The same preprocessed data in Study 1 (Deuchar et al., 2014; Lee et al., 2017) were used to retrieve CS words. As noted previously, a CS word is defined as a word in a different language than its preceding word within a sentence. For example, in the sentence below, which also appears in Table 1, the words “apply,” “那个,” and “job” are all considered CS words. When retrieving CS words from the Spanish-English corpus, as in Study 1, words were considered only when both the word and its preceding word within a sentence had unambiguous language identities. For the Mandarin-English corpus, we retrieved 1,827 unique CS words in Mandarin and 3,716 in English. For the Spanish-English corpus, there were 1,613 unique CS words in Spanish and 2,176 in English.

刚才 我 不是 跟 你 讲 我 apply 那个 job

“Haven’t I told you just now that I applied for that job”

To find the TEs of the CS words, we used Google Translate.^1^ Google Translate is a popular and reliable translation tool based on a neural machine translation model (Wu et al., 2016). The neural machine translation model usually learns from large samples of parallel text and therefore can find the most common translation when given an input. When the input is a single word, Google Translate will output the most likely translation. Note that the most likely translations for some CS words are phrases rather than single words, such as “理工” in Mandarin and “science and technology” as the translation in English. Since this study mainly focuses on CS behaviors at the word level, the CS words translated as phrases were not included in the analysis. All of the CS words and the TEs were lowercased to avoid the same word with and without the capitalized letter being treated differently.

##### Word Frequency

We used the SUBTLEX corpora of Mandarin (Cai and Brysbaert, 2010), Spanish (Cuetos et al., 2012), and English (Brysbaert and New, 2009), which are based on word frequencies from film and television subtitles. CS words and TEs not contained in the SUBTLEX corpora were excluded.

#### Semantic Networks and Word Embedding Models

A weighted semantic network was constructed for each language of each bilingual group. The nodes of a network represent both the CS words and the TEs, whereas the edge weights between every pair of nodes were obtained from the corresponding word embedding models. Unlike Study 1, in this study, the semantic network was built for each language separately. This is because the present study also assesses the properties of the words being replaced (i.e., TEs of the CS words); such properties are likely to be hidden in a CS corpus as not all TEs of the CS words can be found in the bilingual corpus. However, by building separate semantic models based on pre-trained large-scale word embeddings of each language, the semantic properties of TEs can also be obtained.

##### Semantic Vectors

As sublexical features will not affect the analysis of this study and fastText (Bojanowski et al., 2017) is better at representing semantic information than word2vec (Mikolov et al., 2013), we used fastText in Study 2. For each language, we used a pre-trained fastText model^2^ which contains semantic vectors of two million unique words. CS words and TEs that were not covered in fastText models were excluded. Consequently, there were 909 Mandarin-English and 2,839 English-Mandarin CS-TE pairs from the Mandarin-English speech, and 258 Spanish-English and 623 English-Spanish pairs from the Spanish-English speech.

### Analysis

The clustering coefficient of each word in each semantic network was calculated. To determine whether CS words have lower clustering coefficients and higher frequencies than their TEs, we compared the clustering coefficient and the frequency separately between each CS word and its TE, in both CS-TE directions (e.g., for the Mandarin-English bilingual speech: CS words in Mandarin and their TEs in English and CS words in English and their TEs in Mandarin).

#### Data Rescaling

As clustering coefficients and word frequencies of different languages came from different resources, to make the metrics comparable across different languages, we transformed the data to rescale them before the statistical analyses.

##### Clustering Coefficient

We used z-scores to standardize the clustering coefficients of words in different languages. The z-score of a clustering coefficient denotes how many standard deviations it is below or above the mean clustering coefficient of words in its language. It rescales data so that clustering coefficient distributions of two languages have the same mean and standard deviation (*μ* = 0, *σ* = 1).

##### Frequency

We used frequencies measured per million words as standardized word frequency values across languages, which are provided in the SUBTLEX corpora (Brysbaert and New, 2009; Cai and Brysbaert, 2010; Cuetos et al., 2012). In addition, because word frequency values are positively skewed, we log transformed them.

#### Analysis Plan

We first evaluated the performance of Google Translate. Although we used Google Translate to find the best matching translation in general, we could not guarantee that the translation was correct given the context in the sentence. To evaluate how well it reflects actual semantic meanings of words produced in the corpus, we retrieved 10 subsamples from the translations, with each subsample containing 30 CS-TE pairs with the corresponding sentences that the CS words came from. For each subsample, we evaluated the translation accuracy; namely, the percentage of CS-TE pairs that correctly captured the context of the sentences out of all 30 CS-TE pairs. The evaluations were done for both bilingual groups.

Next, we analyzed the correlation between clustering coefficients and frequencies of words for each language in each bilingual group. To distinguish the effect of clustering coefficient from the effect of word frequency, we calculated the residuals of each of these variables in the following statistical analyses to remove the effect of the other variable. For example, when testing the effect of clustering coefficient with word frequency being controlled for, residualized standardized clustering coefficients (Res *C_Z_*) were calculated from a regression model using frequency as the predictor of clustering coefficient. Similarly, residualized log-transformed frequencies (Res *LogF*) were calculated with clustering coefficients being controlled for when testing the frequency effect.

Finally, we conducted both parametric and non-parametric analyses to test the effect of Res *C_Z_* or Res *LogF* on bilingual CS. The parametric test considers the difference values, whereas the non-parametric test only counts the direction of the difference. As the non-parametric test has been shown to be less powerful than the parametric one (Olejnik and Algina, 1987), we use the non-parametric test as backup evidence while still primarily relying on the parametric test for interpreting the results.

Mixed-design ANOVAs and sign tests were adopted as parametric and non-parametric tests, respectively. With either Res *C_Z_* or Res *LogF* as the dependent measurement, a mixed-design ANOVA was run using a within-item variable of switching (whether the word was a CS word or a TE), and a between-item variable of CS-TE direction. Paired-sample *t*-tests were used to further analyze the switching effect in each CS-TE direction in the case of significant interactions. For each CS-TE direction, the sign test was also run based on counts of CS-TE pairs with lower versus higher Res *C_Z_* or Res *LogF* for CS words.

### Results

#### Evaluating Google Translate

The average accuracy of the translations was 0.81 (*SD* = 0.04) for the Mandarin-English words and 0.77 (*SD* = 0.07) for the Spanish-English words.

#### Correlation Between Dependent Measurements

For the CS words and the TEs from the Mandarin-English corpus, the standardized clustering coefficient and the log-transformed word frequency were positively correlated (*r* = 0.51, *p* < 0.001). For the words from the Spanish-English corpus, the correlation between the two variables was also positive (*r* = 0.55, *p* < 0.001).

#### The Effect of Clustering Coefficients on CS

**Figure 5:**
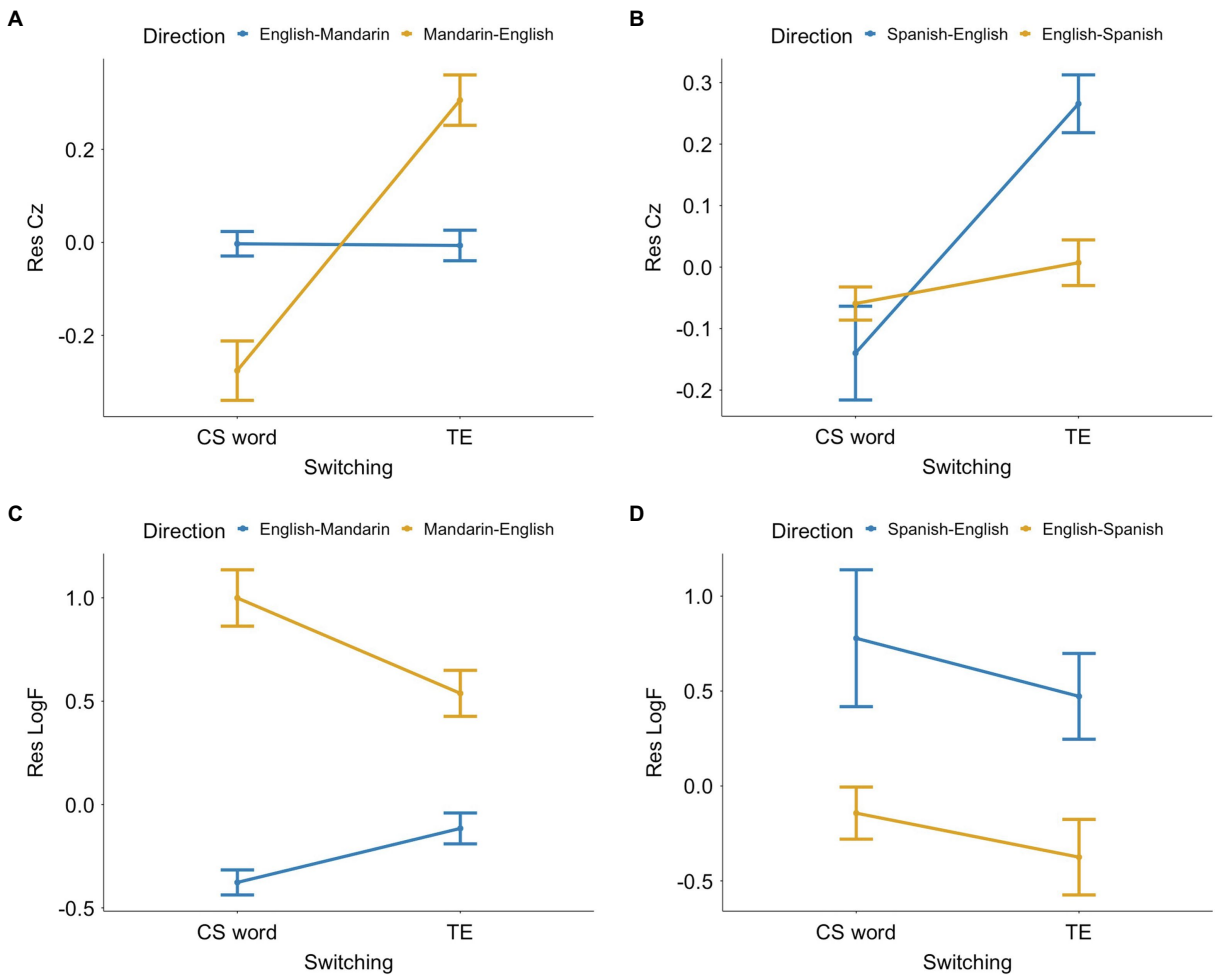
Means for switching and CS-TE direction of the residualized standardized clustering coefficients (Res *C_Z_*) for **(A)** the Mandarin-English corpus and **(B)** the Spanish-English corpus, and the residualized log-transformed frequencies (Res *LogF*) for **(C)** the Mandarin-English corpus and **(D)** the Spanish-English corpus. The error bars represent the 95% confidence intervals.

For the data from the Mandarin-English corpus, the mixed-design ANOVA showed a significant interaction between switching and CS-TE direction (Figure 5A), *F*(1, 3746) = 177.45, *p* < 0.001. Paired-sample *t*-tests revealed that Res *C_Z_* of CS words were significantly lower than their TEs in the Mandarin-English direction, *t*(908) = −13.84, *p* < 0.001, *d* = −0.46. However, the difference was not significant in the English-Mandarin direction.

For the data from the Spanish-English corpus, the interaction between switching and CS-TE direction was also significant (Figure 5B), *F*(1, 879) = 19.74, *p* < 0.001. Paired-sample *t*-tests showed a significant difference between Res *C_Z_* of CS words and TEs, and consistent with the Mandarin-English data, the difference was significant only when the CS-TE direction was from Spanish to English, where CS words had significantly lower Res *C_Z_* than their TEs, *t*(257) = −5.25, *p* < 0.001, *d* = −0.33.

The sign tests indicated significantly more CS-TE pairs that had lower Res *Z_C_* for their CS words than their TEs. That was true regardless of the bilingual group or CS-TE direction. Detailed statistical output is presented in Table 4.

**Table 4:** Statistical results of the sign tests for the clustering coefficient of words.

Bilingual group	CS-TE direction	Count (*C_CSword_ < C_TE_*)	Count (*C_CSword_ > C_TE_*)	*χ^2^*
Mandarin-English	Mandarin-English	595	314	86.87^***^
	English-Mandarin	1511	1328	11.80^***^
Spanish-English	Spanish-English	148	110	5.60^*^
	English-Spanish	387	236	36.60^***^

*C_CSword_ < C_TE_ represents the number of CS-TE pairs in which the CS word had a lower residualized standardized clustering coefficient (Res C_Z_) than its TE, whereas C_CSword_ < C_TE_ represents the opposite pattern*.

* *p < 0.05*;

*** *p < 0.001*.

#### The Effect of Frequencies on CS

For the data from the Mandarin-English speech, the mixed-design ANOVA showed a significant interaction between switching and CS-TE direction (Figure 5C), *F*(1, 3746) = 93.35, *p* < 0.001. Paired-sample *t*-tests showed that Res *LogF* of CS words were significantly higher than their TEs when CS-TE direction was from Mandarin to English, *t*(908) = 6.74, *p* < 0.001, *d* = 0.22. On the English-Mandarin direction, however, CS words had significantly lower Res *LogF* than their TEs, *t*(2838) = −7.22, *p* < 0.001, *d* = −0.14.

For the data from the Spanish-English speech, the interaction between switching and CS-TE direction was not significant (Figure 5D). However, the main effect of switching was significant such that CS words had significantly higher Res *LogF* than their TEs, *F*(879) = 14.07, *p* < 0.001. Paired-sample *t*-tests indicated significantly higher Res *LogF* of CS words than their TEs in both the Spanish-English direction, *t*(257) = 2.44, *p* = 0.015, *d* = 0.15, and the English-Spanish direction, *t*(622) = 3.04, *p* = 0.002, *d* = 0.12.

In sign tests, only the Mandarin-English direction and the English-Spanish direction showed significantly more CS-TE pairs with higher Res *LogF* in CS words. The English-Mandarin direction showed significantly fewer CS-TE pairs with higher Res *LogF*, whereas no significant difference was found in the Spanish-English direction. Detailed statistical output is presented in Table 5.

**Table 5:** Statistical results of the sign tests for the frequency (per million) of words.

Bilingual group	CS-TE direction	Count (*F_CSword_ < F_TE_*)	Count (*F_CSword_ > F_TE_*)	*χ^2^*
Mandarin-English	Mandarin-English	358	551	40.98^***^
	English-Mandarin	1519	1320	13.95^***^
Spanish-English	Spanish-English	131	127	0.06
	English-Spanish	223	400	50.29^***^

*F_CSword_ < F_TE_ represents the number of CS-TE pairs in which the CS word had a lower residualized log-transformed frequency (Res LogF) than its TE, whereas F_CSword_ > F_TE_ represents the opposite pattern*.

*** *p < 0.001*.

### Discussion

For both bilingual groups, we found an effect of clustering coefficients when CS involved using the non-dominant language to replace the dominant language. More specifically, the CS words tend to have lower clustering coefficients than their TEs. However, when CS occurred in the opposite direction, such an effect was not detected. The results are consistent in that the Mandarin-English and the Spanish-English bilingual groups reveal similar patterns. With word frequencies controlled for, we also ruled out the possibility that the influence of clustering coefficient is merely a byproduct of the traditional frequency effect.

The current study also showed opposing effects of clustering coefficients and word frequencies on the CS path from the non-dominant to the dominant language, that is, CS words tend to have lower clustering coefficients but higher word frequencies than their TEs. Such findings not only align well with the previous studies (Gollan and Ferreira, 2009; Gross and Kaushanskaya, 2015) in which word frequencies affect language choice during voluntary CS, but also suggest that the clustering coefficient contributes to bilingual language processing independently of word frequencies.

Consistent with the previous empirical studies of voluntary CS (Gollan and Ferreira, 2009; Gross and Kaushanskaya, 2015), Study 2 showed that when the dominant language is code-switched to the non-dominant language, the mechanisms involved may be different than when CS is in the other direction, from the non-dominant to the dominant language. Following Gross and Kaushanskaya’s (2015) reasoning, such asymmetry might indicate that CS behaviors cannot be purely explained by clustering coefficients and word frequencies. Clustering coefficients in the English to Mandarin or English to Spanish directions were not significantly different between the CS words and their TEs. Word frequencies, however, were significantly different between CS words and their TEs; they showed opposite effects between the two bilingual groups according to the parametric tests.

The non-parametric tests revealed some disparity from the results of the parametric test. For clustering coefficients, the non-parametric tests also found significantly more CS-TE pairs with lower clustering coefficients in CS words than their TEs in the CS-TE direction from the dominant language to the non-dominant language, which further strengthens the effect of clustering coefficients on CS. For word frequencies, the non-parametric tests failed to capture the difference between CS words and TEs in Spanish-English as indicated by the parametric test, which raises a caveat for interpreting the word frequency effect.

One alternative way of defining CS words might be to differentiate between the matrix language and the embedded language (e.g., Auer and Muhamedova, 2005) and only treat words from the embedded language as CS words. For example, in the Mandarin-English CS sentence “刚才 我 不是 跟 你 讲 我 apply 那个 job” (see Code-Switching words and TEs), Mandarin could be considered the matrix language with English as the embedded language, since Mandarin is the language shaping the syntactic structure of the sentence. Unlike in our study, one could then argue that the word “那个” is not a CS word. However, there has been little empirical evidence suggesting that the switch from the embedded language back to the matrix language does not, psychologically, involve a “switch” process. In fact, almost all previous word-level CS experiments defined a switching condition as occurring when the target word is in a different language from its preceding word (e.g., Gollan et al., 2014; Kleinman and Gollan, 2016; de Bruin et al., 2018). The matrix and embedded language distinction focuses more on the syntactic structure of CS behaviors. Additionally, there have been disagreements about how to distinguish between matrix and embedded language (Auer and Muhamedova, 2005; Zabrodskaja, 2009). This is also an issue for our data as not all sentences clearly show which language is the matrix language. For example, in the Mandarin-English CS sentence “就是 那个 路 然后 lead to heritage center” (meaning “it is that road that then leads to heritage center”), the Mandarin and the English words are equally important in determining the sentence structure (i.e., subject, verb, and object). Given that, the current study adopts a less contentious, more easily applied definition of CS. Nevertheless, future studies should closely examine mechanisms and mental processes involved in spontaneous CS speech.

## A Further Investigation of the Language Distance Effect

Although the two bilingual groups showed similar patterns in most analyses, they greatly differ in the frequency of CS; the intra-sentential CS speech accounts for a much smaller portion of the total speech in the Spanish-English data than in the Mandarin-English data. One potential account underlying such differences across bilingual groups might be the language distance effect (Abutalebi et al., 2015; Kim et al., 2016; Ramanujan, 2019a,b). Since English is closer to Spanish than to Mandarin, at both the lexical and the syntactic levels (Voegelin and Voegelin, 1977), Spanish-English bilinguals might use more cognitive control to avoid cross-language conflicts.

To explore whether this is the case, in this study, we conducted an analysis based on preliminary evidence from cross-language cognates. Cognateness between Spanish and English is a lexical-level factor known to greatly reduce the distance between Spanish and English and therefore one that might affect CS speech. If cognateness is a driving force for fewer occurrences of intra-sentential CS, we should expect that the greater the similarity between the word produced and its TE is, the less likely that either of the two words would be used as a CS word. This analysis was done only on the Spanish-English group data as cognateness does not exist for the Mandarin-English group.

### Methods

We first retrieved all words produced in the corpus. A word was assigned to the CS class if it was ever used as a CS word. Otherwise, it was assigned to the non-CS class. Corresponding TEs for those words were then obtained through the same method described in Section “Code-Switching Words and TEs.” Next, we used the orthographic similarity measurement provided by the NIM database (see Guasch et al., 2013 for the detailed algorithm) to measure cognateness between the word and its TE. A logistic regression was then calculated using orthographic similarity to predict whether a word was in the CS or the non-CS class.

### Results

The logistic regression analysis showed orthographic similarity to be negatively related to the probability that the word was a CS word, *B* = −0.46, *Wald* = 16.13, *p* < 0.001. However, the regression only accounted for 0.5% of the variance in the outcome, $R_{c\&s}^{2}$ *=* 0.005. The results indicate that there was a weak effect of cognateness on CS speech such that the greater the orthographic similarity between the word produced and its TE in the other language, the less likely that the word was used as a CS word.

## General Discussion

The study of CS speech has had a long tradition and has produced a wealth of knowledge in the understanding of bilingual language processing and representation. Most studies in this area, however, have focused on a small set of CS words due to the constraints of experimental tasks or available data. In addition, few studies have examined the global structural properties of the CS words. In the current research, we adopted a novel network science approach to overcome these limitations to investigate bilingual semantic lexicons. Specifically, the network science framework has the advantage of depicting structural properties of complex systems, thus allowing us to understand the overall organization of bilingual lexicons as well as the detailed interconnections of words in the two languages. This framework offers us the flexibility to examine the overall properties of the CS words in one language as compared with their non-CS counterparts (i.e., TEs in the other language). Moreover, compared to approaches used in the previous bilingual research in psycholinguistics, the network science approach can be applied directly to the analysis of spontaneous speech in naturalistic conversations, which is of great value when a growing number of studies nowadays have recognized the situation-dependent diversity of language processing (Blanco-Elorrieta and Pylkkänen, 2017; Kałamała et al., 2020).

Based on analyses of two CS corpora of naturalistic productions, our studies showed the following patterns. First, the semantic organizations of the two lexicons in CS speech are largely distinct, with a small portion of overlap such that the semantic network community dominated by each language still contains words from the other language. The lexicon of the non-dominant language shows more dependence on the lexicon of the dominant language, which is reflected in the network community measures: The community of the non-dominant language is more open to words from the other language, compared to the community of the dominant language.

This finding aligns well with the Competition Model that posits modular representations for different languages that emerge out of lexical competition (Bates and MacWhinney, 1982; Hernandez et al., 2005; Hernandez, 2013). Moreover, much as in the previous work (Hernandez et al., 2005; Li and Zhao, 2013), the greater dependence of the non-dominant language reveals that the lexicon of the non-dominant language can be parasitic on the lexicon of the dominant language (Hernandez et al., 2005; Li and Zhao, 2013). The lexicon of the dominant language can be independent and integrated by itself to a great extent, but the lexicon of the non-dominant language tends to rely on and overlap with the dominant language at least partially. Although the parasitic lexical-semantic organization in the Li and Zhao (2013) model is due to factors, such as age of acquisition and language proficiency, the current study shows that such organization could also be reflected in the global network structure of the lexicon in spontaneous CS productions. Future studies could use the network science approach to further explore the characteristics of the dominant language’s words that reside in or become parasitic in the lexicon of the non-dominant language. On the other hand, since there are a few words from the non-dominant language that “intrude” into the lexicon of the dominant language, it may also be important to understand the particular properties allowing them to do so.

Second, the effect of clustering coefficients on CS speech when the CS word is in the non-dominant language is consistent across the two bilingual groups and is independent of the word frequency effect. Our findings also underscore the importance of studying words in a global structure that incorporates the interconnection and the interaction between words in the bilingual context. As an essential metric in network science, the clustering coefficient has been shown to be important in other areas of language processing but has not yet been carefully examined in the bilingual literature. For example, Chan and Vitevitch (2009, 2010) showed that words with lower clustering coefficients were more easily retrieved than words with higher clustering coefficients. Following their rationale, it is likely that a word with a lower clustering coefficient will “stick out” from other neighboring words, whereas a word with a higher clustering coefficient tends to be overwhelmed by its densely interconnected neighbors. Therefore, bilinguals can retrieve the word with lower C more easily, although the cost of doing that is the necessity to switch between languages. Combining this finding with the observed separate language modules in Study 1, it is likely that the lexical accessibility driven by the interconnectivity of words makes the cost-free switching between modules possible; a word with lower clustering coefficient might be able to “stick out” and surpass the competitive interplay between the two languages. To test this possibility, a more direct link between the language modules and the overriding lexical accessibility should be established in the future.

Such findings also add to the work of Chan and Vitevitch (2009, 2010) on phonological networks. With modern semantic embedding tools, which have been found to accurately capturing the semantic representations of words, we showed that the effect of clustering coefficients on language processing can also be identified in semantic domains and bilingual contexts. However, the current research is unable to tell us whether the underlying mechanism of the clustering coefficient in semantic domains is comparable to the mechanism uncovered by Chan and Vitevitch (2009, 2010) in phonological networks. To deepen our understanding of the clustering coefficient in semantic networks, future studies should combine experimental methods and computational modeling with the network science approach.

Third, Spanish-English bilinguals switched less frequently than Mandarin-English bilinguals. One likely explanation might be the language distance effect (Abutalebi et al., 2015; Kim et al., 2016; Ramanujan, 2019a,b). We found that the greater the orthographic similarity between the word produced and its TE, the less likely that the word is used as a CS word. As Ramanujan (2019b) showed that two typologically similar languages require greater cognitive control than two languages whose linguistic attributes have greater disparities, Spanish-English bilinguals might use more cognitive control to avoid cross-language conflicts and therefore mix languages less frequently than Mandarin-English bilinguals during speech. The occasional intra-sentential CS sentences with mixed lexicons might just be evidence of how the two languages resemble each other lexically and syntactically. However, orthographic similarity only accounts for a very small portion of CS behaviors, perhaps because we are examining naturalistic CS productions rather than written data. Phonological similarity might be a better predictor than orthographic similarity in speech studies, and phonological measurements can also be applied to Mandarin-English bilingual data. In addition, although bilinguals in the two corpora have almost equally high proficiency in both languages (Deuchar et al., 2014; Lee et al., 2017), their language backgrounds and the environments of data collection may still differ in other ways, as various linguistic and situation-dependent factors could affect CS behaviors (e.g., Li, 1996).

The present research highlights the importance of considering diversity of different bilingual groups in language research. If it is the distance between the two languages that affects bilingual CS speech and the relevant semantic organizations of bilingual lexicons, then a language distance effect should also be observed in other bilingual groups. Future studies should test this conjecture by analyzing bilinguals whose language pairs represent various degrees of linguistic distance.

Finally, as noted above, CS is affected by whether the CS word is in the dominant or non-dominant language, with different effects of clustering coefficients and word frequencies depending on CS-TE direction (i.e., dominant to non-dominant or the reverse). This finding suggests that the word frequency effect that has been observed in some previous CS studies might be modulated by other factors, such as language dominance or the distance between the two languages. It is also likely that the interactions between word frequency, language dominance, and language distance account for why word frequency effects on CS were mixed in the previous studies (Gollan and Ferreira, 2009; Gross and Kaushanskaya, 2015; de Bruin et al., 2018). Investigation of the complex relationships among these variables will be important in future research.

In Study 2, the semantic representation was built separately for each language. The consideration for doing so, as mentioned before, was to uncover the effects of clustering coefficients of the words being replaced (i.e., TEs of CS words). However, it has been widely argued that a bilingual is not the sum of two monolinguals (Grosjean, 1989). Therefore, we caution that the separate semantic representations as constructed in Study 2 might not best represent the actual bilingual situation. To overcome this limitation, a larger bilingual CS corpus or word association norms in bilingual context might help in modeling bilingual semantic representations. Although Google Translate shows reliable performance in general (Wu et al., 2016), its accuracy in reflecting the context-dependent meanings of words in the present study still needs to be improved. Future work should explore the use of context-dependent translation for words or recruit human translators. In addition, due to the methodological limitations of corpus analysis, the current study provides correlational rather than causal findings. As always, caution should be used in interpreting correlational analyses as evidence for causal relationships. Future studies should combine corpus analyses with experimental methodologies to identify causal explanations of spontaneous bilingual CS speech. Finally, the current study has examined only clustering coefficients and word frequencies in CS speech. Future studies should investigate other lexical variables known to affect speech production, such as word length (Piantadosi et al., 2011), phonological overlap (Costa et al., 2005), and phonological neighborhood density (Gahl and Strand, 2016).

## Data Availability Statement

The raw data supporting the conclusions of this article will be made available by the authors, without undue reservation.

## Author Contributions

QX: research design, data analysis, and paper writing. MM and MC: data analysis and paper writing. PL: research design and paper writing. All authors contributed to the article and approved the submitted version.

## Conflict of Interest

The authors declare that the research was conducted in the absence of any commercial or financial relationships that could be construed as a potential conflict of interest.

## Publisher’s Note

All claims expressed in this article are solely those of the authors and do not necessarily represent those of their affiliated organizations, or those of the publisher, the editors and the reviewers. Any product that may be evaluated in this article, or claim that may be made by its manufacturer, is not guaranteed or endorsed by the publisher.

## Footnotes

^1^The translation was automatically done by the Google Translate API for Python. https://github.com/lushan88a/google_trans_new

^2^ https://fasttext.cc/docs/en/crawl-vectors.html
