# Corrigendum: Modeling Bilingual Lexical Processing Through Code-Switching Speech: A Network Science Approach

**DOI:** 10.3389/fpsyg.2021.816653

**Published:** 2021-12-15

**Authors:** Qihui Xu, Magdalena Markowska, Martin Chodorow, Ping Li

**Affiliations:** ^1^Department of Psychology, Graduate Center, The City University of New York, New York, NY, United States; ^2^Department of Linguistics, Institute for Advanced Computational Science, Stony Brook University, Stony Brook, NY, United States; ^3^Department of Psychology, Hunter College, The City University of New York, New York, NY, United States; ^4^Department of Chinese and Bilingual Studies, Faculty of Humanities, The Hong Kong Polytechnic University, Hong Kong, China

**Keywords:** code-switching speech, bilingual lexicon, network Science, community detection, clustering coefficient, computational linguistics

In the original article, there was a mistake in the legend of *Figure 3* as published. Figure 3 has a labeling error. The correct legend appears below.

**FIGURE 3 |** Proportions of words within each community in Mandarin-English **(A,B)**, and Spanish-English **(C,D)**. Networks are based on all sentences and word2vec embeddings. For the Spanish-English plots, “Other” represents words with ambiguous language identities.

The authors apologize for this error and state that this does not change the scientific conclusions of the article in any way. The original article has been updated.

## Publisher's Note

All claims expressed in this article are solely those of the authors and do not necessarily represent those of their affiliated organizations, or those of the publisher, the editors and the reviewers. Any product that may be evaluated in this article, or claim that may be made by its manufacturer, is not guaranteed or endorsed by the publisher.

